# Immediate release fentanyl in general practices: Mostly off-label prescribing

**DOI:** 10.1080/13814788.2023.2165644

**Published:** 2023-01-25

**Authors:** Yvette M. Weesie, Liset van Dijk, Marcel L. Bouvy, Karin Hek

**Affiliations:** a Netherlands Institute for Health Services Research (Nivel), Utrecht, The Netherlands; b Department of PharmacoTherapy, -Epidemiology & -Economics (PTEE), Faculty of Science and Engineering, Groningen Research Institute of Pharmacy, University of Groningen, Groningen, The Netherlands; c Utrecht Institute for Pharmaceutical Sciences, Utrecht University (UU), Utrecht, The Netherlands

**Keywords:** General practices, immediate release fentanyl, off-label

## Abstract

**Background:**

The immediacy of the onset of opioids may be associated with the risk of dependency and accidental overdose. Nasal and oromucosal fentanyl dosage forms are so called immediate release fentanyl (IRF). These IRFs have been approved to treat breakthrough pain in patients with cancer who are on chronic opioid treatment only. There are signals of increased off-label prescribing of IRFs in general practices.

**Objectives:**

This study aims to provide insight into the frequency of IRF prescription in Dutch general practices and the extent to which IRF is prescribed off-label.

**Methods:**

Routinely collected electronic health records of general practices (GPs) participating in Nivel Primary Care Database were used. Adult patients with IRF prescriptions in 2019 were selected from whom dispensing data on 2018 and 2019 was available. Diagnoses were recorded by GPs using International Classification of Primary Care. Descriptive analyses were performed.

**Results:**

This study included 342 GPs with a patient population of 1,297,942 patients, 1,368 patients received at least one IRF prescription in 2019, which is equal to 1.1 patients per 1,000 registered patients. Most patients (74.9%) with an IRF prescription received an off-label prescription. A slight majority had a cancer diagnosis but nearly 65.2% did not have a maintenance therapy and 14% were opioid-naive before receiving their first IRF prescription.

**Conclusion:**

IRFs are not prescribed frequently in Dutch general practices. However, when prescribed, a relatively large portion of patients received an off-label prescription.

KEY MESSAGES

- Immediate release fentanyl (IRF) is when prescribed, often prescribed off-label in general practices.
- Most patients with an off-label prescription were not on a maintenance treatment before or during their IRF prescription.
- There is room for improvement for GPs when prescribing IRFs, especially about prescribing an IRF only in combination with maintenance opioid treatment.

## Introduction

Fentanyl is a widely used opioid in the Western world and is 50–100 times more potent than morphine [1–5]. It is prescribed for chronic pain via patches or breakthrough pain via immediate release forms such as nasal and oromucosal administration forms, also known as immediate release fentanyl (IRF). IRF produces rapid analgesia, which can closely match the time of an episode of breakthrough pain. IRF has been shown to provide effective treatment for breakthrough pain [6,7]. However, the use of IRF may have an increased risk of abuse, especially to patients without a cancer diagnosis [4,8,9]. Moreover, IRF has an improved bioavailability and a more rapid onset of respiratory depressant effects increasing the risk of unintentional overdose [8]. IRF has been linked to an increase in drug overdose deaths in the United States and European countries [10,11]. And even though reports of IRF prescribing in European countries are low, there are signs of an increase in IRF prescriptions [1,12].

The European Medicines Agency (EMA) approved IRF for breakthrough pain in adult cancer patients who are already on maintenance treatment of at least 60 Morphine Milligram Equivalent (MME). According to the Summary of Product Characteristics (SmPC) IRF should not be prescribed to opioid-naive patients, patients with severe obstructive lung conditions or other indications than breakthrough pain. All other use is defined as off-label. Off-label use is not illegal and sometimes even appropriate but it also brings clinical, safety and ethical issues [13,14], because specific off-label use has often not been thoroughly studied yet. An association between off-label prescribing and a higher risk of adverse drug events was found, even when corrected for patient and drug-related characteristics [15]. Also, drugs can be safe for adults but when used by children, can cause significant adverse events [16]. For example, off-label use of fentanyl appeared to be more frequently associated with respiratory depression, both age and indication, when used by children [17].

Most studies on fentanyl are performed in the hospital setting or clinical trial. Yet, general practitioners (GPs) are usually the primary health care provider for patients with chronic pain and are more often confronted with dilemmas where they have to choose to prescribe IRF to a patient or not [18,19]. These patients are usually not included in clinical trials and are generally more complex than patients selected for clinical trials or other studies [20,21]. Therefore it is essential to look at data that reflect daily clinical real-world practice.

This study aims to provide insight into the frequency of IRF prescription in Dutch general practices, and the extent to which IRF is prescribed off-label.

## Methods

### Data source

Data used in this study were derived from the Nivel Primary Care Database (Nivel-PCD), which includes routine care data originating from electronic medical records from about 500 general practices across the Netherlands. The participating GPs constitute a representative sample of the total population of Dutch GPs, approximately 10% [22,23]. Within the Dutch health care system, all residents are mandatorily registered with only one general practice that keeps track of the patient’s complete medical record and whose GPs fulfil a gatekeeper role for access to medical specialists. The Nivel-PCD database consists of patient characteristics (age, sex) and longitudinal information on GPs consultations, diagnoses and drug prescriptions. Diagnoses are recorded by GPs using the International Classification of Primary Care version 1 (ICPC-1). Prescriptions are coded using the Anatomical Therapeutic Chemical Classification system (ATC) and contain all prescriptions registered in the GPs information system. This may also include prescriptions from medical specialists that pharmacies dispense. The study period covered 2019.

Dutch law allows the use of these data for research purposes under certain conditions. According to this legislation, neither obtaining informed consent from patients nor approval by medical ethics committee is obligatory for observational studies containing no directly identifiable data (Dutch Civil Law, Article 7:458). This study has been approved by the applicable governance bodies of Nivel-PCD under nr. NZR00320.014.

### Study population

We selected general practices with sufficient quality prescription data in Nivel-PCD in 2018 and 2019. The data quality is determined by a set of requirements, including weeks of registration (at least 46 weeks of data in one year), sufficient registration of meaningful prescriptions registered with ATC-codes (at least 85% of all prescriptions) and at least 75% of all consultations need at least one diagnoses registered for that patient on the day of consultation. We then selected patients who had at least one IRF prescription in 2019 and were registered in 2018 and 2019. Data from 2018 were used to establish if a patient was opioid naïve in 2019. IRF prescriptions were defined as prescriptions with ATC-code N02AB03 (fentanyl) and a nasal, oromucosal or sublingual form of administration.

### Off-label prescribing

Prescriptions were labelled as off-label when an IRF: (1) was prescribed for an indication other than cancer, (2) when patients did not have an opioid maintenance prescription of 60 MME and/or (3) when IRF was prescribed to a patient younger than 18.

#### Indication

The official indication for an IRF is breakthrough pain in cancer patients. However, there is no ICPC-code for breakthrough pain. Therefore we looked at all ICPC-codes concerning cancer. Because there is not a single ICPC code for cancer, an overall code was computed, including all ICPC codes regarding cancer diagnoses (Appendix). Indications registered with the prescription can have missing values therefore, we also include indications registered with consultations to determine whether a patient has a cancer diagnosis.

#### Other indications

Other indications were calculated on ICPC-1 chapter level and individual ICPC-codes. Per patient, only the unique diagnoses were counted. For example, if a patient has five IRF prescriptions for low back pain (L03), it will count as one patient with an IRF prescription for low back pain. It is possible that a patient has more than one unique diagnosis, for example, low back pain (L03) and shoulder symptom/complaint (L08). All of the unique diagnoses per person were counted.

#### Maintenance treatment

Maintenance treatment is defined in the SmPC as prescriptions with a morphine milligram equivalent (MME) of 60 or higher, prescribed at least one week prior to the IRF prescription. To determine whether or not the prescription was off-label, we looked at the first IRF prescription in 2019. If needed, we used data from 2018 to assess if the first 2019 IRF prescription was preceded by maintenance treatment. An IRF was considered part of the maintenance treatment when the IRF was prescribed within 120 days after the last opioid. The MME value of the prescriptions was determined by multiplying the prescribed regimen (for instance, once a day, one tablet), with the strength of the opioid determined by the product number registered with the prescription. When a range was given in the prescribed regimen, for example, 1–2 tablets a day, the mean value was used for calculating MME. If a prescription was prescribed ‘as needed’, the mean value (combining zero and the maximum dosage registered with the prescription) was also used in the calculation. Being on a maintenance treatment is reported as a yes or no variable or unsure. A maintenance treatment is labelled unsure when the prescribed regimen or other information concerning the strength of the opioid is missing from the prescription. A patient may have more IRF prescriptions in 2019, however, only the first prescription in 2019 was used to determine off-label prescribing concerning maintenance therapy.

### Contraindications

The following contraindications were included in the analysis: (1) being an opioid naïve patient, defined as a patient who had no opioid prescription in the year prior to the IRF, (2) patients under the age of 18 years and (3) patients who had at least one consultation for severe obstructive lung conditions (measured as having COPD or asthma (ICPC R95, R96)) in 2019.

### Number of IRF prescriptions

To indicate how many prescriptions patients received during the study period (2019), we looked at the total number of IRF prescriptions per patient.

### Analysis

Descriptive analyses were conducted to describe the frequency and nature of IRF prescribing in Dutch general practice. The rate of IRF prescribing was expressed as the number of patients with at least one IRF prescription per 1,000 patients-years. Patient-years were based on the part of the year in which a patient was registered with their GP, in quartiles. If a patient was only registered for half a year, the patient was counted as 0.5 within the population instead of 1. Off-label prescribing, patient characteristics and contraindications were expressed in percentages of patients with at least one IRF prescription.

## Results

This study included 342 GPs with a population of 1,297,942 patients representing 1,273,108 patient-years, 1,368 patients received at least one IRF prescription in 2019, which is equal to 1.1 patients per 1,000 registered patient-years.

**Figure 1.**
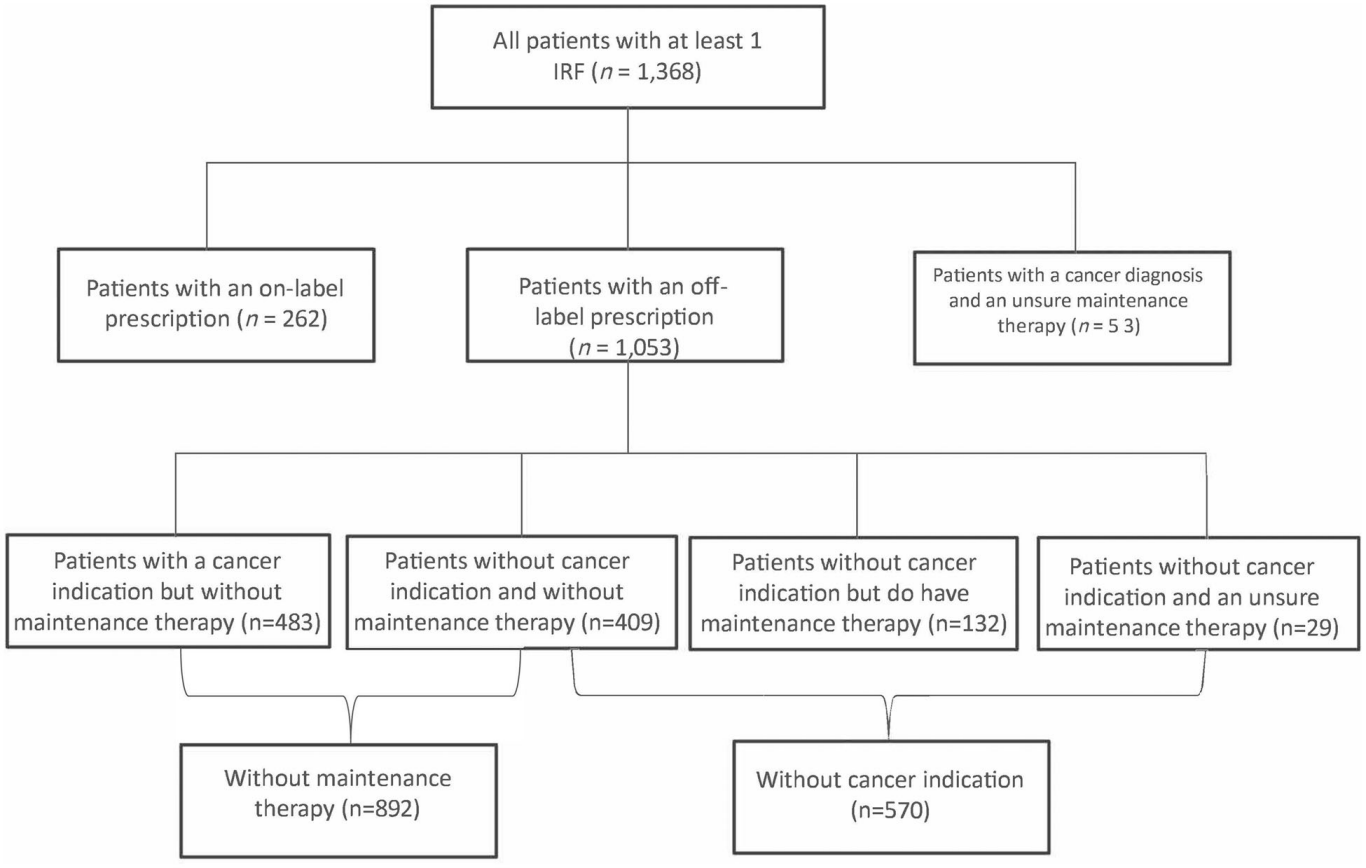
Flowchart of patients who were prescribed an immediate release fentanyl (IRF) in 2019.

Table 1 shows the patient characteristics of patients prescribed at least one IRF in 2019, 58.7% were female and more than half of the patients receiving an IRF were 70 years or older. Figure 1 shows that most patients (*n* = 1,053) received their prescription off-label, equal to 0.7 per 1,000 registered patient-years. More than half (*n* = 892, 65.2%) of all patients with at least one IRF were not on maintenance therapy and 41.7% (*n* = 570) did not have a cancer indication, 409 (30.0%) patients met neither of the requirements.

**Table 1.** Patient characteristics of patients who at least received one IRF in 2019.

	Patient with at least 1	Patients without an IRF	Total patient population
	IRF (*n* = 1,368)	(*n* = 1,296,574)	(*n* = 1,297,942)
Sex	*n* (%)	*n* (%)	*n* (%)
Male	565 (41.3)	642,504 (49.6)	643,069 (49.6)
Female	803 (58.7)	654,070 (50.5)	654,873 (50.5)
Age category			
<18 years	1 (0.1)	233,580 (18.0)	233,581 (18.0)
18–49 years	122 (8.9)	502,684 (38.8)	502,806 (38.7)
50–59 years	179 (13.1)	198,770 (15.3)	198,949 (15.3)
60–69 years	282 (20.6)	168,639 (13.0)	168,921 (13.0)
70–79 years	343 (25.1)	126,407 (9.8)	168,921 (9.8)
80–89 years	305 (22.3)	56,178 (4.3)	56,483 (4.4)
90 + years	136 (9.9)	10,316 (0.8)	10,452 (0.8)

Table 2 shows the prevalence of contra-indications for IRF in 2019, 16% of the patients with an IRF had respiratory comorbidity and 14% of the patients were opioid naive. There was only one patient who was younger than 18.

**Table 2.** Prevalence of contra-indications in patients with an IRF.

	*N* (%)
Respiratory comorbidities	
COPD	153 (11.2)
Asthma	63 (4.6)
COPD and asthma	3 (0.2)
No asthma or COPD	1,149 (84.0)
Opioid naive	
Yes	191 (14.0)
No	1,177 (86.0)

Table 3 shows the number of IRF prescriptions per patient in 2019 for those who got prescribed IRF. Most patients (56.0%) received only 1 IRF prescription in 2019 but 14% received six or more IRF prescriptions in 2019. Patients who received an off-label prescription received slightly more IRFs than patients who had on-label prescriptions.

**Table 3.** Number of prescriptions, by category off-label per medication episode.

	Off-label	On-label	Total^a^
Number of prescriptions			
1 Prescription	600 (57.0)	127 (48.5)	766 (56.0)
2–3 Prescriptions	233 (22.1)	79 (30.2)	320 (23.4)
4–5 Prescriptions	58 (5.5)	26 (9.9)	86 (6.3)
6 Or more prescriptions	162 (15.4)	30 (11.5)	196 (14.3)
Mean number of prescriptions (SD)	3.4 (5.2)	2.8 (3.2)	3.2 (4.9)

^a^Also include patients with unknown off/on-label prescription.

Most IRF prescriptions were prescribed for cancer indications. When IRFs were prescribed for other indications, musculoskeletal indications were most common (22.1%), followed by general and unspecified (8.6%) and digestive (6.7%). Zooming in on specific diagnoses, low back pain with radiation (5.8%), fractures: other (3.6%) and pain general/multiple sites (3.5%) were most common.

## Discussion

### Main findings

Approximately 1.1 per 1,000 registered patient-years received an IRF prescription in Dutch general practices in 2019. Most patients (77.0%) with an IRF prescription received this prescription off-label, 65.2% of the patients were not on a maintenance treatment and 14% was opioid-naive before receiving their first IRF prescription. A small majority of patients with an IRF had a cancer diagnosis (58.3%) but a large proportion of that group did not receive a proper maintenance treatment (60.5% of patients with a cancer diagnosis).

Our study confirms previous findings that IRF prescribing is low in European countries [24,25]. After all, IRF should be reserved as a last resort for breakthrough pain in cancer. Although this relatively low use, the steep increase in IRF prescribing that has also been observed in other countries may be worrying. A Spanish study showed a 74% increase in IRF prescribing over the period of 2012–2017 (from 3.9 to 6.8 per 10,000 patients). A French study showed an increase of 263% in the prescription of transmucosal fentanyl between 2006 and 2015 and another showed that one-third of the patients in the period 2007–2019 received an off-label transmucosal fentanyl prescription [1,12,26]. This indicates that more insight into prevalence, treatment and reasons to prescribe IRF is needed.

In a population of around 17 million people, like in the Netherlands, these study results show that an estimated total of nearly 14 thousand patients received an off-label IRF prescription in 2019. This could have serious safety concerns for patients. The high potency and rapid brain entry increases the risk of serious harm to patients and makes this type of opioid prone to abuse [4,8]. We found that nearly 15% of the patients received six or more IRF prescriptions. This is not corresponding to recommendations in the official product information, IRF prescriptions should be used as rescue medication for breakthrough pain. If a patient has to use a series of IRFs, it indicates an unbalanced maintenance therapy which should be adjusted instead of getting prescribed another IRF [27].

The lack of maintenance treatment and being opioid-naive can have serious safety consequences because of the high risk of respiratory depression, even at low doses in patients without proper opioid tolerance [9]. IRF should never be used as a first prescription due to serious associated risks and limited benefits [8]. And even though there was only one patient under 18, it is still important to be aware of the risks. Paediatric opioid pharmacokinetic data are highly variable [28,29]. Although several clinical trials suggest some benefits of IRF over placebo or other opioids in non-malignant pain [6,7], the general attitude is very reluctant towards the use of IRF for non-malignant pain [18,19]. Especially when the study in trials reporting benefits is highly selective and consists of patients with low risk for adverse events. Furthermore, the duration of these studies is often short. This is an entirely different setting than the real-world general practice setting in our study. Like previous studies in this setting, we see that IRF prescriptions are prescribed for other diagnoses than breakthrough pain in cancer, for example, for the musculoskeletal system [8,24]. More research on fentanyl prescribing and its effects is necessary in non-selective patient populations. And more general practice settings should be taken into account, instead of RTC’s in hospital settings, because of the complex patient population and the fact that GPs are often primary caregiver to patients with chronic pain conditions [18,19].

### Strengths and limitations

A strength of our study is that we used a representative dataset including a patient population of about 10% of the Dutch population. We combined diagnoses with prescriptions and gave detailed information on patient characteristics of patients who receive an off-label prescription in real-world situations.

A limitation of our study is that we could not use a specific ICPC-code for breakthrough pain as such a code does not exist. Therefore, we cannot specify whether the IRF is prescribed for breakthrough pain in cancer or breakthrough pain in any other chronic conditions. By including all cancer diagnoses as on-label, we might have underestimated the prevalence of off-label prescribing. Moreover, we cannot specify if the IRF prescriptions in the other diagnoses were prescribed for breakthrough pain in chronic non-cancer pain. Another limitation is that missing data are common because we use data registered in daily clinical practice. It is possible that, for example, an instruction registered with the prescription could differ slightly from the instruction given to the patient verbally. Also, there was missing data on diagnoses in 31.1% of the patients. This can happen when, for example, the GP receives feedback from the pharmacy that a prescription is prescribed by someone other than the GP or when a prescription is prescribed repeatedly. And lastly, we see all prescriptions registered within the GP information system. These include prescriptions from medical specialists that pharmacies dispense. A negligible proportion of prescriptions from specialists may be missing in the system. Therefore, we could both slightly overestimate the problem of missing maintenance prescriptions but also slightly underestimate the overuse of IRF. We do not expect this to influence our findings substantially.

### Clinical implications

The use of IRF is increasing in Europe [1,12]. Therefore awareness of its risks is important. Many patients did not receive a maintenance therapy at the start of the IRF. The risks of respiratory depression in opioid-naive patients or patients without maintenance therapy is high [4]. Monitoring of patients with any opioid prescription is crucial but when prescribing IRF it is essential to take into account the higher risk of abuse and safety concerns [8].

## Conclusion

This study showed that the prevalence of prescribing IRF in Dutch general practices is low, but when prescribed, off-label prescribing is considerably high. Especially, a considerable proportion of patients do not receive proper maintenance therapy. Awareness of the potential risks of IRF should be increased in the general practices.

## Appendix

**Table:**

ICPC-1 code	
A79	Malignancy NOS
B72	Hodgkin’s disease/lymphoma
B73	Leukaemia
B74	Malignant neoplasm blood other
D74	Malignant neoplasm stomach
D75	Malignant neoplasm colon/rectum
D76	Malignant neoplasm pancreas
D77	Malignant digestive neoplasm, other/NOS
L71	Malignant neoplasm musculoskeletal
N74	Malignant neoplasm nervous system
R84	Malignant neoplasm bronchus/lung
R85	Malignant neoplasm respiratory other
S77	Malignant neoplasm of skin
T71	Malignant neoplasm thyroid
U75	Malignant neoplasm of kidney
U76	Malignant neoplasm of bladder
U77	Malignant neoplasm urinary other
W72	Malignant neoplasm related to pregnancy
X75	Malignant neoplasm cervix
X76	Malignant neoplasm breast female
X77	Malignant neoplasm genital female other
Y77	Malignant neoplasm prostate
Y78	Malignant neoplasm male genital other
